# Detailed corneal and genetic characteristics of a pediatric patient with macular corneal dystrophy - case report

**DOI:** 10.1186/s12886-021-02041-y

**Published:** 2021-07-23

**Authors:** Anna Nowińska, Edyta Chlasta-Twardzik, Michał Dembski, Ewa Wróblewska-Czajka, Klaudia Ulfik-Dembska, Edward Wylęgała

**Affiliations:** 1 Chair and Clinical Department of Ophthalmology, Faculty of Medical Sciences in Zabrze, Medical University of Silesia in Katowice, ul. Panewnicka 65, 40-760 Katowice, Poland; 2 Ophthalmology Department, Railway Hospital in Katowice, Katowice, Poland

**Keywords:** Macular corneal dystrophy, Confocal microscopy, Optical coherence tomography, CHST6, Corneal dystrophy, Cornea

## Abstract

**Background:**

Corneal dystrophies are a group of rare, inherited disorders that are usually bilateral, symmetric, slowly progressive, and not related to environmental or systemic factors. The majority of publications present the advanced form of the disease with a typical clinical demonstration. The initial signs and symptoms of different epithelial and stromal corneal dystrophies are not specific; therefore, it is very important to establish the early characteristic corneal features of these disorders that could guide the diagnostic process.

**Case presentation:**

The main purpose of this study was to report the differential diagnosis of a pediatric patient with bilateral anterior corneal involvement suspected of corneal dystrophy. An 8-year-old male patient presented with asymptomatic, persistent, superficial, bilateral, diffuse, anterior corneal opacities. Slit lamp examination results were not specific. Despite the lack of visible stromal involvement on the slit lamp examination, corneal analysis based on confocal microscopy and optical coherence tomography revealed characteristic features of macular corneal dystrophy (MCD). The diagnosis of MCD was confirmed by *CHST6* gene sequencing. The early corneal characteristic features of MCD, established based on the findings of this case report, include corneal astigmatism (not specific), diffuse corneal thinning without a pattern of corneal ectasia (specific), and characteristic features on confocal microscopy (specific), including multiple, dark, oriented striae at different corneal depths.

**Conclusions:**

The clinical examination should be complemented with corneal imaging techniques, such as confocal microscopy and optical coherence tomography. In patients suspected of corneal dystrophy, genetic testing plays an important role in establishing the final diagnosis.

## Background

Macular corneal dystrophy (MCD; OMIM #217,800; ORPHA98969) is a stromal corneal dystrophy indicated as category 1 according to the International Committee for Classification of Corneal Dystrophies (IC3D) (Category 1: A well-defined corneal dystrophy in which the gene has been mapped and identified and specific mutations are known) [1]. The first symptoms of the disease are usually reported in the second and third decade of life and include gradually deteriorating vision, photophobia, tearing and, rarely, recurrent epithelial erosions [2, 3].

The majority of publications and ophthalmic atlases present the advanced form of the disease with a typical clinical demonstration of diffuse, stromal haze and gray-white corneal opacities involving the entire corneal stroma from limbus to limbus; therefore, the diagnosis of dystrophy in the early stages could be challenging. Moreover, diversity in the clinical presentation of MCD has been reported in recent years. Patients with predominantly deep stromal lesions in the peripheral cornea or isolated Descemet membrane deposits have been reported, and the diversity of presentations was confirmed in an optical coherence tomography study [4–6]. Additionally, the disease is of an autosomal recessive origin, expressed only in individuals homozygous for a mutation, which usually causes the diagnosed patient to become the first one affected in the family. In contrast, autosomal dominant corneal dystrophies, such as epithelial–stromal transforming growth factor beta-induced (TGFBI) corneal dystrophies, usually affect many family members, which facilitates the diagnosis of a child from a family with a known diagnosis.

MCD is characterized by a low prevalence. It is estimated that as an indicator, MCD accounts for less than 1 % of keratoplasties according to the Australian Corneal Graft Registry (ACGR) and to the Eye Bank Association of America (EBAA) [7, 8]. In the USA, the prevalence of MCD was calculated as 9.7 per million individuals [9]. While MCD is relatively rare in the United States, it is reported to be most prevalent in Iceland, India and Saudi Arabia [10–12]. Despite its rarity, among stromal dystrophies, MCD is described as the most common [9–11]. Nevertheless, due to the generally rare prevalence of stromal dystrophies, the differential diagnosis of MCD may be challenging, especially in the early stages of the disease.

The main pathogenetic factor of MCD is the progressive intra- and extracellular accumulation of glycosaminoglycans in stromal keratocytes, Descemet membranes and endothelial cells, leading to the loss of transparency in the corneas of affected patients [1–3, 13]. Mutations in the carbohydrate sulfotransferase gene (*CHST6*), encoding corneal N-acetylglucosamine-6-sulfotransferase (C-GlcNac6ST), have been linked to MCD and further reported in patients originating from populations of different regions throughout the world. There is mutational heterogeneity and the predominance of missense mutations, accounting for approximately 50 % of MCD cases) whereas nonsense mutations, deletions, insertions or indels account for approximately 30 % of MCD cases [3, 14–16].

Historically, the confirmation of the diagnosis of MCD was based on the presence of basophilic granular material, which stains positively with periodic acid Schiff, Alcian blue, metachromatic dyes and colloidal iron intra- and extracellularly within the corneal stroma, Descemet membrane and endothelial cells [1]. The broad introduction of noninvasive corneal imaging techniques, including confocal microscopy and anterior segment optical coherence tomography (AS OCT), has revolutionized the diagnosis process and has had a great impact on understanding the pathophysiology of corneal dystrophies.

Confocal microscopy is widely used for analyzing the in vivo microscopic structure in several clinical conditions, such as infectious keratitis, dry eye syndrome, corneal dystrophies and degenerations [17, 18]. Characteristic features of advanced MCD, which have been described and connected to structural and histopathologic data, include hyperreflective areas of basal epithelial cells, rectilinear hyperreflective areas in the anterior stroma, a specific granular appearance to keratocytes and the extracellular matrix accompanied by dark striae of different lengths and orientations, and polymegethism of the corneal endothelial cells containing bright granules in their cytoplasm [19, 20].

AS OCT enables in vivo cornea and other anterior chamber structure imaging with an axial resolution from 18 μm with time domain OCT (TD OCT) and 5.0 μm with spectral domain OCT (SD OCT) to 2,0 μm with high definition OCT (HD OCT). The main pathological features of advanced MCD described in previous studies include general increased reflectivity throughout the corneal stroma, irregularity of the anterior stromal border from the epithelium side, diffuse areas of hyperreflectivity in Bowman’s layer caused by corneal deposits and a noticeable layer of increased reflectivity in the posterior, peripheral corneal part [5, 20–22].

For patients with a suspicion of corneal dystrophy, the diagnostic flow includes a detailed familial history, the onset and characterization of signs and symptoms as revealed on the slit lamp exam, and corneal morphology and topography analysis using an available imaging system, such as optical coherence tomography, Scheimflug imaging, Orbscan or confocal microscopy. Finally, molecular diagnosis is becoming increasingly important for establishing the correct diagnosis in challenging cases, as is the development of novel genotype-specific treatments.

Considering the challenges in corneal dystrophy differential diagnosis, especially in pediatric populations, we would like to report a pediatric case of macular corneal dystrophy in which the final diagnosis was established based on detailed corneal imaging data confirmed by genetic testing. The case report is part of a study approved by the Ethics Committee of the Medical University of Silesia, Katowice, Poland (KNE/0022/KB1/43/I/14; 01.07.2014). The patients’ parents signed an informed consent form before any study procedure.

## Case presentation

An 8-year-old male patient was referred to the Chair and Clinical Department of Ophthalmology, School of Medicine with the Division of Dentistry in Zabrze, Medical University of Silesia in Katowice by a general ophthalmologist due to persistent, superficial corneal opacities revealed when diagnosed with the mild conjunctivitis two months earlier. The conjunctivitis was treated with ofloxacin eye drops (3 mg/ml) four times daily and resolved after one week. No specific testing, such as cell culture, immune assays or polymerase chain reaction (PCR), was performed at the acute stage of the conjunctivitis. Due to the persistent superficial corneal abnormalities in both eyes and a suspected diagnosis of multiple subepithelial corneal infiltrates (MSIs) caused by epidemic keratoconjunctivitis (EKC), the patient was administered hydrocortisone eye drops (3.35 mg/ml) twice daily for two weeks and once daily for one week. There was no significant difference in corneal status; therefore, the patient was referred to our clinic. On examination, he reported no ocular complaints. He had no significant medical or drug history, as well as no previous ocular disease in either eye, except for the reported conjunctivitis. The patient did not have a relevant family history.

**Fig. 1:**
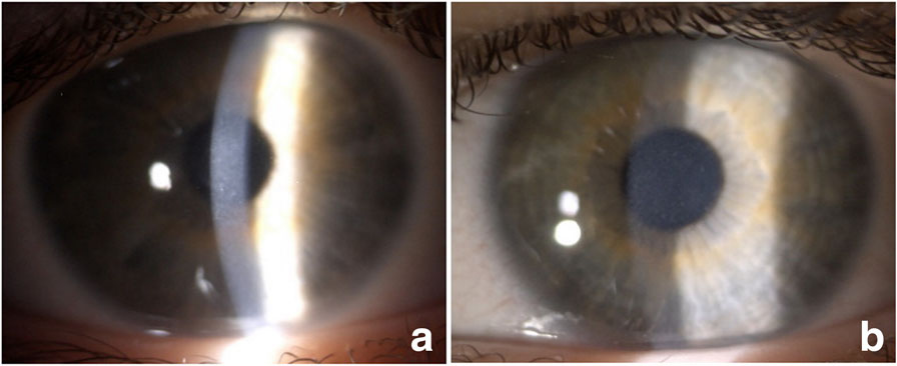
Representative slit-lamp photographs (SL 9900, Haag-Streit type, CSO, Italy; magnification 10x) of the right eye (**a**) and left eye (**b**). Multiple, diffuse, grayish, indistinct, superficial corneal opacities accompanied by subepithelial haze are visible. The severity of the corneal changes was comparable between the eyes

The best corrected visual acuity (BCVA) was right eye (RE) (cc + 6.0 Dsph − 4.5 Dcyl axis 170°) 1.0; left eye (LE) (cc + 6.0 Dsph − 3.5 Dcyl axis 175°) 1.0. The ocular refraction after tropicamide cycloplegia (1 % tropicamide eye drops administered 3 times every 15 min) was RE, + 7.0 Dsph − 4.75 Dcyl axis 170°; LE, + 7.0 Dsph − 4.0 Dcyl axis 175°. The intraocular pressure (IOP) in both eyes was 14 mmHg. The axial length was 20.94 mm for the right eye and 21.12 mm for the left eye as measured by an IOL Master (Carl Zeiss Meditec, Inc., Dublin, California, USA). On slit lamp examination, multiple diffuse, grayish, indistinct, superficial corneal opacities extending from limbus to limbus accompanied by subepithelial haze were present. The severity of the corneal changes was comparable between eyes (Fig. 1). Fundus examination was unremarkable.

**Fig. 2:**
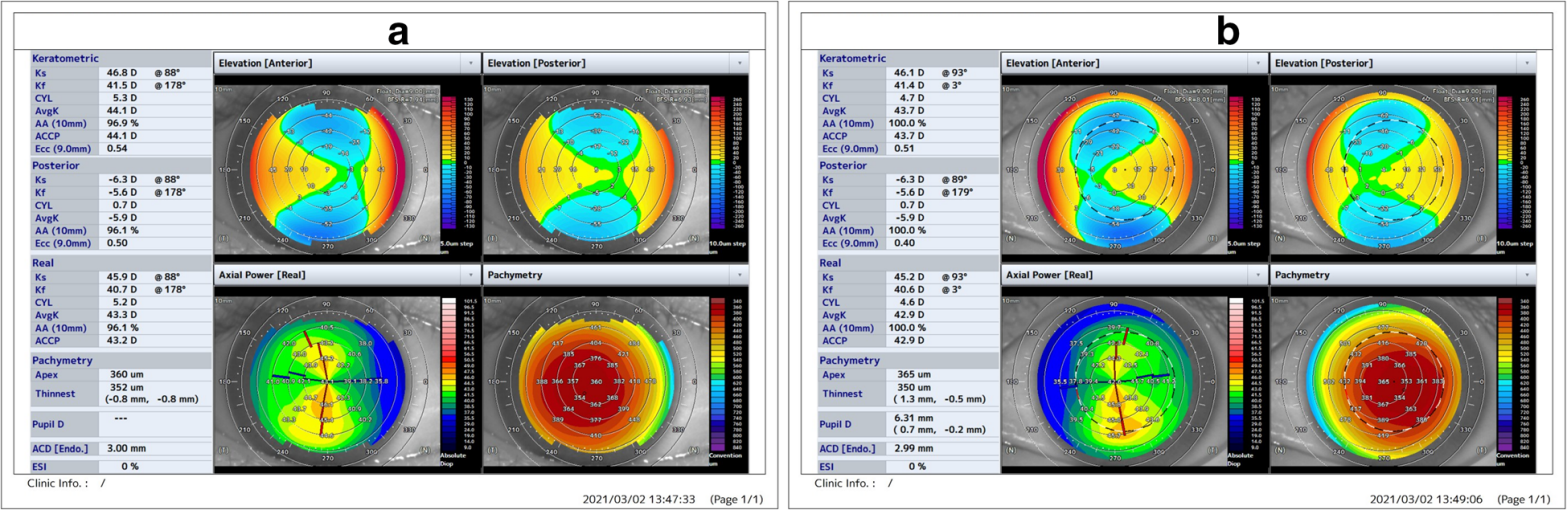
Swept source OCT (SS OCT) corneal map mode results (16 radial B-scans with 800 A scans per line sampling, total scan duration 0.3 s, diameter 16 mm, and scan depth 11 mm) showing the summary of the keratometric, posterior and real keratometry data. Elevation anterior and posterior, real axial power and pachymetry maps of (**a**) the right eye and (**b**) the left eye.Pachymetry map revealed bilateral, general corneal thinning. The location of the thinnest corneal point was the inferotemporal region in both eyes. The keratometric map confirms the presence of with-the-rule astigmatism. The corneal shape was characterized as prolate, both of the anterior and posterior surfaces. Anterior and posterior elevation maps showed a typical pattern in which the apex demonstrated warm colors above the best fit sphere (BFS) (RE: 7 μm, 5 μm; LE: 9 μm, 4 μm, respectively) with a slightly elevated isthmus that joined the central cornea from the temporal periphery. Right-left mirror symmetry was present. The Ectasia Screening Index (ESI), which is a parameter used in the detection of corneas with ectasia patterns implemented in SS OCT software, was 0 % in both eyes

**Fig. 3:**
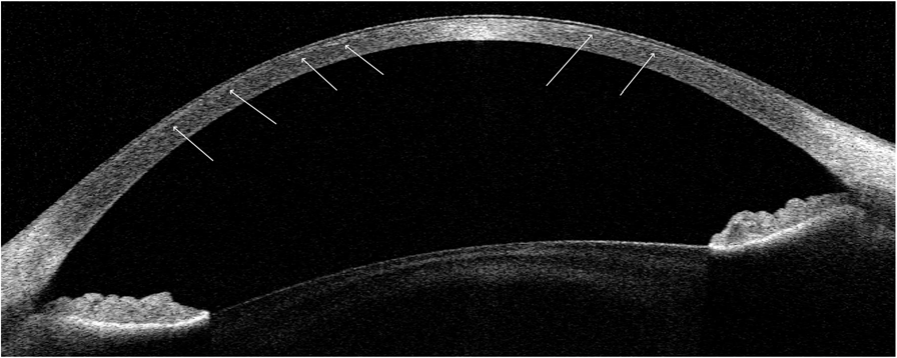
High-definition morphology SS OCT scan. Very discrete, diffuse, superficial hyperreflective opacities with no clearly distinguishable borders and various shapes are visible. Deposits were located in the anterior cornea, just beneath the epithelium (arrows)

Anterior segment swept source OCT (SS OCT; OCT CASIA2; Tomey, Nagoya, Japan) revealed abnormalities regarding the anterior topography and pachymetry map, as did select parameters from Fourier indices analysis. The pachymetry map revealed bilateral, general corneal thinning. The corneal apical thickness (CAT) of the right eye was 360 μm, and that of the left eye was 365 μm. The thinnest portions of the cornea were 352 and 350 μm thick, respectively. The location of the thinnest corneal point was the inferotemporal region in both eyes. Keratometric and posterior topography maps confirmed the presence of with-the-rule astigmatism (RE = 5,3 D; LE = 4,7 D). The corneal shape was characterized as prolate, both for the anterior and posterior surfaces (the anterior and posterior eccentricity of the corneal curve (Ecc) within 9.0 mm was 0.54 and 0.5 in the RE and 0.51 and 0.4 in the LE, respectively). Anterior and posterior elevation maps showed a typical pattern in which the apex demonstrated warm colors above the BFS (RE: 7 μm, 5 μm; LE: 9 μm, 4 μm, respectively) with a slightly elevated isthmus that joined the central cornea from the temporal periphery. Right-left mirror symmetry was present. Three and six millimeter Fourier indices analysis revealed abnormalities regarding two parameters: regular astigmatism on the anterior and posterior surfaces and asymmetry on the anterior surface. The results of 6 mm keratometric and real higher-order index analysis were borderline in both eyes. The Ectasia Screening Index (ESI), which is a parameter used in the detection of corneas with ectasia patterns implemented in SS OCT software, was 0 % in both eyes. The results of the above anterior and posterior corneal surface analyses and the pachymetry map are presented in Fig. 2. High definition (HD) morphology scans of both eyes revealed very discrete, diffuse hyperreflective opacities with no clearly distinguishable borders and various shapes. Deposits were located in the anterior cornea, just beneath the epithelium (Fig. 3).

**Fig. 4:**
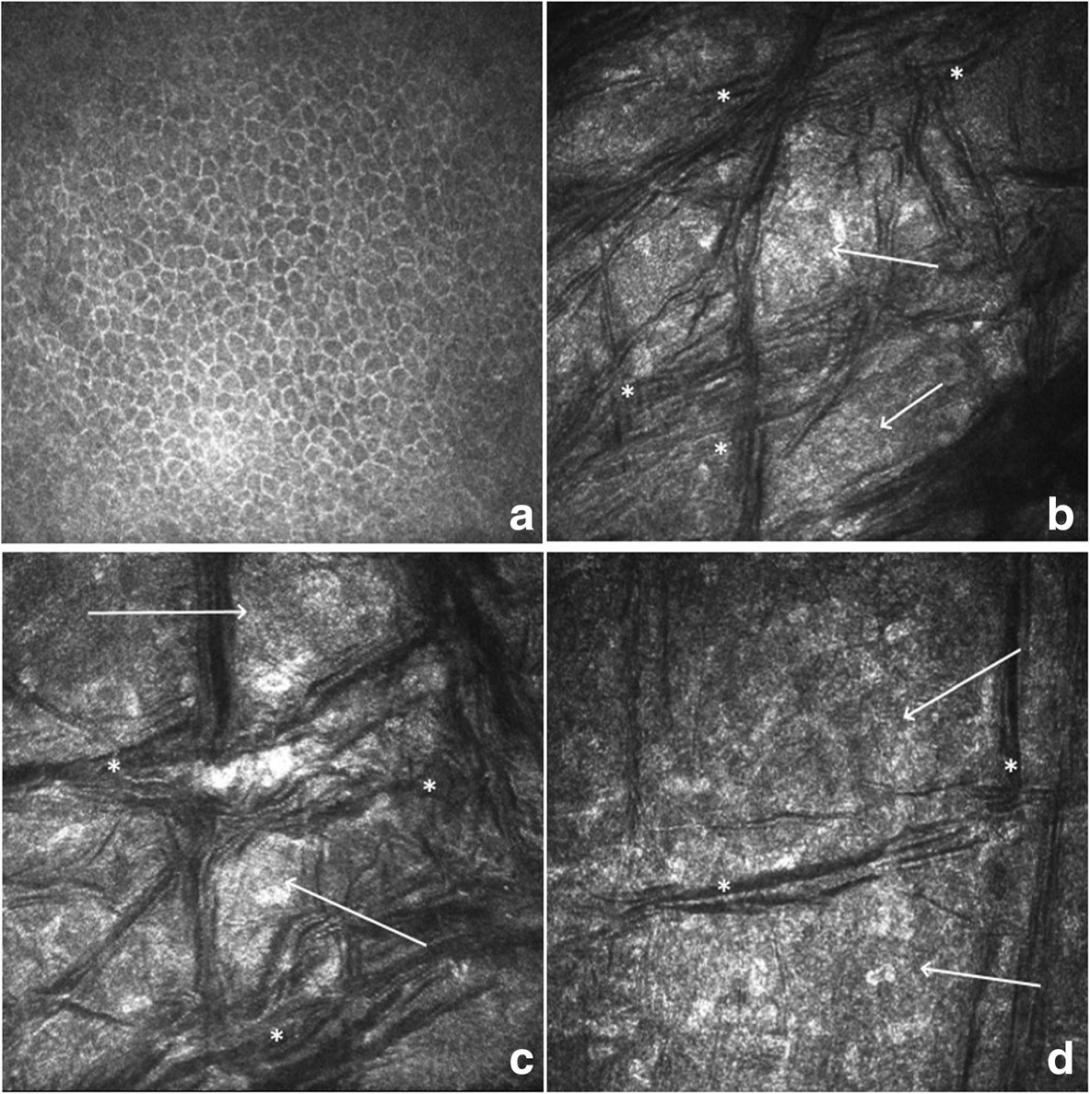
Representative confocal microscopy images at different scanning depths. (**a**) Confocal image at a depth of 10 μm. No significant abnormalities in the corneal epithelium were visible. (**b**) Confocal image at a depth of 80 μm. (**c**) Confocal image at a depth of 120 μm. (**d**) Confocal image at a depth of 330 μm. Granular, hyperreflective appearance of the corneal stroma (arrows) with multiple, differently oriented dark striae (*). The striae were especially prominent, multilayered, dark and thick at depths from 90–150 μm (**b**, **c**), becoming less evident at increasing depths (**d**)

A Heidelberg Retina Tomograph 3 Rostock Cornea Module (Heidelberg Engineering GmbH, Dossenheim, Germany) was used for the in vivo assessment of the detailed corneal morphology after topical instillation of 0.5 % proparacaine hydrochloride (Alcaine, Alcon Laboratories, Fort Worth, TX, USA) eye drops. The epithelial layers were of normal morphology. Starting at the level of Bowman’s layer, multiple, differently oriented dark striae were visible. The morphology of the striae changed at different corneal depths. The striae were especially prominent, multilayered, dark and thick at depths of 90–150 μm, becoming less evident at increasing depths. The keratocytes and the intercellular space had a granular, hyperreflective appearance throughout the whole stroma. The corneal endothelial morphology was not documented due to poor cooperation of the child during this invasive, uncomfortable examination. The results of confocal microscopy are presented in Fig. 4. In contrast to the slit lamp and OCT examination results, where the pathological changes were localized to the superficial layers of the cornea, confocal microscopy revealed changes affecting the whole corneal stroma.

Based on the corneal morphology examination, a suspected diagnosis of stromal corneal dystrophy was made. The majority of corneal dystrophies are characterized by familial occurrence; therefore, the parents were carefully examined to identify possible corneal abnormalities. The examination did not reveal any changes. The familial history also did not reveal any significant ocular diseases or parental consanguinity. Accordingly, a diagnosis of autosomal recessive stromal corneal dystrophy, mainly macular corneal dystrophy, was suspected. The patient could also potentially be the first affected member of the family in case the corneal dystrophy is autosomal dominant.

**Fig. 5:**
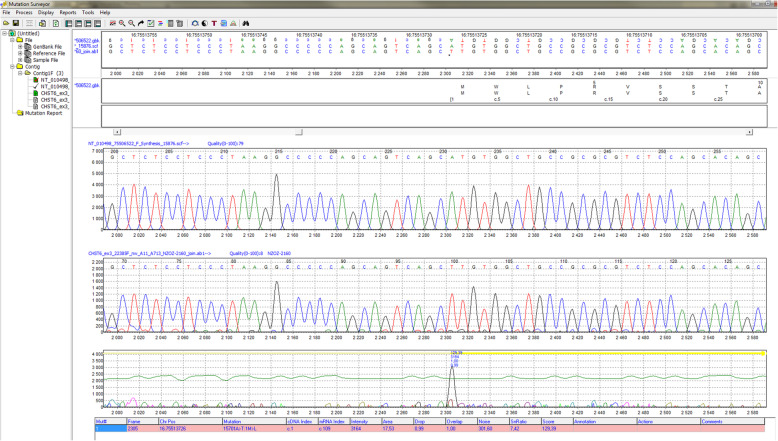
Graphic presentation of the homozygous pathogenic variant c.1 A > T (p.M1?) of the *CHST6* gene

To establish the final diagnosis, next-generation sequencing was incorporated. Blood samples were collected from the patient and patient’s parents after obtaining written informed consent for genetic testing of the coding regions of the following genes linked with corneal dystrophies: *CHST6, TGFBI, KRT3, KRT12, COL8A2, SLC4A11, TACSTD2, UBIAD1, VSX1*, and *ZEB1*. The next-generation sequencing (NGS) technique was performed with the SeqCap EZ Hyper Cap protocol and a NimbleGen SeqCap EZ probe kit (Roche Sequencing Solutions, Inc; CA; USA) using a NextSeq sequencer by Illumina (Illumina, San Diego, CA, USA). NGS analyses with a mean read coverage of at least 40× were considered successful. The test result was confirmed using the Sanger reference method, described in detail in our previous publication [20]. All mutations were described according to the Human Genome Variation Society nomenclature (HGVSv19.01). For the analysis of the *CHST6* gene, the reference sequence with accession number NM_021615.4 (HGMD Professional 2019.4) was used. The known homozygous, pathogenic variant c.1 A > T (p.M1?) was found following *CHST6* gene analysis for our patient (proband). *CHST6* gene sequencing for both parents revealed the same variant in a heterozygous state (Fig. 5).

Based on the results of the corneal morphology analysis combined with the genetic testing results, the diagnosis of macular corneal dystrophy was made. The patient was referred to the corneal outpatient clinic for further control visits.

## Discussion and csonclusions

Corneal dystrophies, according to the IC3D classification system are categorized into epithelial and subepithelial, epithelial–stromal TGFBI, and stromal and endothelial dystrophies [1]. The overall classification includes 22 different types of corneal dystrophies, frequently further divided into subcategories. The differential diagnosis of corneal dystrophies is challenging and includes infectious corneal diseases, mostly of the viral form, corneal degenerations, such as mosaic (crocodile shagreen) degeneration, cornea farinata or peripheral hypertrophy, subepithelial corneal degeneration, vernal keratoconjunctivitis, and keratopathies related to dry eye disease, such as keratoconjunctivitis sicca or keratopathies related to blepharitis. Additionally, corneal diseases of unknown origin, such as Thygeson’s superficial punctate keratitis (TSPK), or of various systemic causes, including several skin diseases, monoclonal gammopathy, enzyme-related deficiencies (tyrosinemia, lecithin-cholesterol-acyltransferase deficiency, mucopolysaccharidoses), systemic lysosomal storage diseases or cystinosis, should be incorporated into the differential diagnosis.

After the initial interview and slit lamp examination of our patient, which revealed multiple, diffuse, grayish, indistinct, superficial corneal opacities extending from limbus to limbus, accompanied by subepithelial haze (Fig. 1), the most likely differential diagnoses included epithelial, subepithelial or stromal corneal dystrophy, multiple subepithelial corneal infiltrates caused by epidemic keratoconjunctivitis or Thygeson’s superficial punctate keratitis (TSPK). The majority of epithelial and epithelial–stromal TGFBI corneal dystrophies are autosomal dominant, but the patient could have been the first affected member of the family. Moreover, the early signs of stromal dystrophies include epithelial and subepithelial corneal abnormalities, subsequently followed by stromal involvement; therefore, stromal dystrophies could not be ruled out only because slit lamp examination did not reveal stromal involvement and due to the young age of the patient.

Multiple subepithelial corneal infiltrates may be caused by epidemic keratoconjunctivitis, they usually occur during the subacute and chronic phases and may persist for months to years. In the slit lamp examination, the probability of central corneal involvement, namely, involvement of the pupillary zone, is higher than that of peripheral corneal involvement. This was not consistent with our patient’s results, since the corneal changes were diffuse rather than centralized. On confocal microscopy, MSIs are visible as distinct round hyperreflective plaques, accompanied by increased anterior stromal hyperreflectivity and hyperreflective foci and inflammatory cells within the basal epithelium [23, 24].

Based on the corneal appearance on slit lamp examination, TSPK should also be considered in the differential diagnosis. TSPK is a bilateral corneal disease of unknown origin in which factors such as viral infection or the immune reaction to viral infection and allergic reactions have been proposed to play a pathogenic role. Slit lamp examination reveals numerous punctate opacities involving the epithelium and underlining superficial corneal stroma. Confocal microscopy changes are characteristic and include highly reflective deposits with a starburst-like appearance in the superficial and basal epithelial cell layers, an increased number of dendritic cells in the epithelial basal cell layer and the subepithelial nerve plexus, and highly reflective, tiny, needle-shaped material in the anterior corneal stroma [25, 26].

In contrast to MSIs and TSPK features, confocal microscopy analysis for our patient revealed significant stromal involvement. Starting at the level of Bowman’s layer, multiple, differently oriented dark striae were visible. The keratocytes and the intercellular space had a granular, hyperreflective appearance throughout the whole stroma (Fig. 4). These findings are consistent with previous reports describing confocal corneal changes in MCD. In contrast to those at advanced stages, the epithelium layers of our patient were of normal morphology, while in advanced forms, numerous hyperreflective basal epithelial cells among cells of normal morphology were reported [19, 20]. Despite the lack of visible stromal involvement on slit lamp examination, the cornea presented changes involving the deep stromal layers, confirming the high utility of confocal microscopy in the differential diagnosis of corneal dystrophies.

Clinical examination of our patient also showed regular corneal astigmatism and diffuse corneal thinning, which are characteristic features of MCD, confirmed by previous histopathological, Scheimpflug imaging, ultrasound biomicroscopy and OCT studies [5, 20, 27–30]. The diffuse corneal thinning is believed to be a result of the dysregulation of keratan sulfate proteoglycan synthesis or catabolism, which directly influences corneal structure [31]. The above observation leads us to conclude that generalized corneal thinning precedes the progressive loss of corneal transparency. A high-definition corneal scan of our patient revealed very discrete, diffuse hyperreflective opacities with no clearly distinguishable borders and various shapes located in the subepithelial regions. As the disease progresses, OCT scans show hyperreflective stromal corneal deposits with associated thinning of the epithelium over the deposits and characteristic pre-Descemetic peripheral deposits. In some patients, thickening of the Descemet membrane may be noted. In advanced stages of the dystrophy, dense stromal deposits cause an optical shadow in the posterior part of the cornea. Additionally, the progression of deposits in the endothelial cell layer can eventually lead to endothelial decompensation and increased corneal thickness [1, 4, 5, 20–22, 28].

It has been described that the visual function of patients with corneal dystrophies is not only compromised by scattering induced by the corneal opacity but also associated and correlated with higher-order aberrations (HOAs). HOAs of the total cornea and anterior and posterior surfaces were reported to be larger in subjects with stromal corneal dystrophies than in normal control subjects [32, 33]. The analysis of the Fourier indices for our patient revealed abnormalities regarding two parameters: regular astigmatism on the anterior and posterior surfaces and asymmetry on the anterior surface. The results of the 6 mm keratometric and real higher order index analyses were borderline in both eyes, while the results of the 3 mm and 6 mm posterior higher-order index analyses were within the normal range. Yagi-Yaguchi Y. et al. reported that HOAs were increased at the late stage of MCD. Their study group compromised 13 eyes of seven patients with advanced MCD without genetic confirmation; the mean age of the patients was 49.9 ± 5.8 years, and the visual acuity was logMAR 0.48 ± 0.62 [32]. The results cannot be compared directly to our results because of the significant difference in age and visual acuity (our patient’s age was 8 years, and VA was logMAR 0.0). Age has been reported to be strongly correlated with visual function, refraction, astigmatism and HOAs [34]. The borderline results of the 6 mm higher-order keratometric index in our patient may be directly connected to the anterior subepithelial abnormalities revealed by HD OCT scans and may indicate that Fourier index abnormalities occur early in the disease course. On the other hand, such subtle changes on the anterior surface of the cornea are not specific, and the result may be influenced by several factors, such as disturbances of the tear film, patient cooperation during the examination or internal/indoor environmental factors [35, 36]. Therefore, the influence of MCD on Fourier index results should be studied in a larger sample size, and one case report cannot be representative of the population.

There are few case reports on the cooccurrence of keratoconus and MCD [37, 38]. In the study of Dudakova et al., the authors observed anterior, paracentral steepening of the corneal surface, which was graded as keratoconus by Scheimflug integrated software, but without a coexisting ectasia pattern on the posterior corneal surface [28]. The anterior and posterior Ectasia Screening Index (ESI), which is a parameter used in the detection of corneas with ectasia patterns implemented in the SS OCT software, was 0 % in both eyes of our patient. Thus, our findings may serve as evidence of diffuse corneal thinning not associated with an ectasia pattern.

According to the American Society of Ophthalmology guidelines, genetic testing should be offered to patients with clinical findings suggestive of a Mendelian disorder, whose causative gene(s) have been identified [39, 40]. The known, previously described homozygous pathogenic variant, c.1 A > T, with alteration of the start codon (p.M1?) was found following *CHST6* gene sequencing in our patient, thus confirming a diagnosis of MCD. Alterations of the first codon have also been reported in Polish, Czech, German and Turkish populations [20, 41–43]. It is wort mentioning, that in case of MCD, not only missense mutations are pathogenic, but also deletions, insertions or indels, which account for approximately 30 % of MCD cases. In our case, the upstream region of the CHST6 gene was not covered by the gene sequencing, which could be regarded as a study limitation. Genetic testing provides future prospects for implementing gene therapy. Moreover, MCD with precisely identified genetic defects fulfils the prerequisites for attempting clinical gene therapy [44]. Currently, we are faced with new possibilities in future methods of gene therapy, such as clustered regularly interspaced short palindromic DNA repeat (CRISPR) and CRISPR-associated gene (cas) systems of gene editing. Such an approach was already employed in Meesman corneal dystrophy [44–46].

The main importance of this case report is in defining the early characteristic features of MCD, despite the absence of characteristic corneal abnormalities on slit lamp examination. The main limitations are the difficulties in performing high-quality slit lamp and confocal microscopy scans in an 8-year-old patient. Additionally, the influence of environmental factors and tear film disturbances could interfere with the OCT Fourier indices results.

To conclude, the initial signs and symptoms of different epithelial and stromal corneal dystrophies are not specific; therefore, it is very important to establish early characteristic corneal features that could guide the diagnostic process. The clinical examination should be complemented with corneal imaging techniques, such as confocal microscopy and optical coherence tomography. Early corneal characteristic features of MCD, established according to the findings of the case report, include corneal astigmatism (not specific), diffuse corneal thinning without a pattern of corneal ectasia (specific), and those obtained on confocal microscopy (specific), including multiple, dark, oriented striae at different corneal depths. In patients suspected of corneal dystrophy, genetic testing plays an important role in establishing the final diagnosis and may provide encouraging results for future gene therapy.

## Data Availability

All data generated or analyzed during this study are included in this published article.

## Abbreviations

ACGR: Australian Corneal Graft Registry
AS OCT: Anterior segment optical coherence tomography
BCVA: Best corrected visual acuity
CAT: Corneal apical thickness
Cas: Clustered regularly interspaced short palindromic DNA repeat-associated genes
cc: With refractive correction
C-GlcNac6ST: Corneal N-acetylglucosamine-6-sulfotransferase
CHST6: Carbohydrate sulfotransferase
COL8A2: Collagen Type VIII Alpha 2 Chain
cyl: Cylinder
CRISPR: Clustered regularly interspaced short palindromic DNA repeat
D: Diopter
EBAA: Eye Bank Association of America
EBMD: Epithelial basement membrane dystrophy
EKC: Epidemic keratoconjunctivitis
EREDs: Epithelial recurrent erosion dystrophies
ESI: Ectasia Screening Index
GCD: Granular corneal dystrophy
HD OCT: High definition OCT
HOAs: Higher order aberrations
IC3D: International Committee for Classification of Corneal Dystrophies
IOP: Intraocular pressure
KRT3: Keratin 3
KRT12: Keratin 12
LE: Left eye
MCD: Macular corneal dystrophy
MECD: Meesmann corneal dystrophy
MSIs: Multiple subepithelial corneal infiltrates
NGS: Next generation sequencing
PCR: Polymerase chain reaction
RE: Right eye
SD OCT: Spectral domain OCT
SLC4A11: Solute Carrier Family 4 Member 11
SMCD: Subepithelial mucinous corneal dystrophy
Sph: Spherical
SS OCT: Swept source OCT
TACSTD2: Tumor Associated Calcium Signal Transducer 2
TD OCT: Time domain OCT
TGFBI: Transforming growth factor beta-induced
TSPK: Thygeson's superficial punctate keratitis
UBIAD1: UbiA Prenyltransferase Domain Containing 1
VSX1: Visual System Homeobox 1
ZEB1: Zinc Finger E-Box Binding Homeobox 1

## References

[1] Weiss JS, Møller HU, Aldave AJ, Seitz B, Bredrup C, Kivelä T (2015). IC3D classification of corneal dystrophies-edition 2. Cornea.

[2] Aggarwal S, Peck T, Golen J, Karcioglu ZA (2018). Macular corneal dystrophy: A review. Surv Ophthalmol.

[3] Singh S, Das S, Kannabiran C, Jakati S, Chaurasia S (2020). Macular Corneal Dystrophy: An Updated Review. Curr Eye Res.

[4] Zhang W, Kassels AC, Barrington A, Khan S, Tomatsu S, Alkadi T (2019). Macular corneal dystrophy with isolated peripheral Descemet membrane deposits. Am J Ophthalmol Case Reports.

[5] Chaurasia S, Ramappa M, Mishra DK (2019). Clinical diversity in macular corneal dystrophy: an optical coherence tomography study. Int Ophthalmol.

[6] Chaurasia S, Mishra DK (2019). Atypical presentation of macular corneal dystrophy managed by Descemet stripping endothelial keratoplasty. Indian J Ophthalmol.

[7] Williams K, Anne, Keane M, Claire, Coffey N, Elizabeth, Jones VJ, Mills RA, Coster DJ. The Australian Corneal Graft Registry. 2018. https://nla.gov.au/nla.obj-726934474/view.

[8] Eye Bank Association of America. Eye Banking Statistical Report. 2013. 2013. http://restoresight.org/wp-content/uploads/2014/04/2013_Statistical_Report-FINAL.pdf.

[9] Musch DC, Niziol LM, Stein JD, Kamyar RM, Sugar A (2011). Prevalence of corneal dystrophies in the United States: Estimates from claims data. Investig Ophthalmol Vis Sci.

[10] Das AV, Chaurasia S. Clinical Profile and Demographic Distribution of Corneal Dystrophies in India. Cornea. 2020;Publish Ah. 10.1097/ico.0000000000002450. 10.1097/ICO.0000000000002450 32740009

[11] Alzuhairy S, Alkatan HM, Al-Rajhi AA (2015). Prevalence and histopathological characteristics of corneal stromal dystrophies in Saudi Arabia. Middle East Afr J Ophthalmol.

[12] Liu NP, Smith CF, Bowling BL, Jonasson F, Klintworth GK (2006). Macular corneal dystrophy types I and II are caused by distinct mutations in the CHST6 gene in Iceland. Mol Vis.

[13] Lisch W, Weiss JS (2019). Clinical and genetic update of corneal dystrophies. Exp Eye Res.

[14] Vance JM, Jonasson F, Lennon F, Sarrica J, Damji KF, Stauffer J, Pericak-Vance MAKG (1996). Linkage of a gene for macular corneal dystrophy to chromosome 16. Am J Hum Genet.

[15] Akama TO, Nishida K, Nakayama J, Watanabe H, Ozaki K, Nakamura T (2000). Macular corneal dystrophy type I and type II are caused by distinct mutations in a new sulphotransferase gene. Nat Genet.

[16] Liu NP, Dew-Knight S, Rayner M, Jonasson F, Akama TO, Fukuda MN (2000). Mutations in corneal carbohydrate sulfotransferase 6 gene (CHST6) cause macular corneal dystrophy in Iceland. Mol Vis.

[17] Erie JC, McLaren JW, Patel SV (2009). Confocal Microscopy in Ophthalmology. Am J Ophthalmol.

[18] Kokot J, Wylęgała A, Wowra B, Wójcik Ł, Dobrowolski D, Wylęgała E (2018). Corneal confocal sub-basal nerve plexus evaluation: a review. Acta Ophthalmol.

[19] Micali A, Pisani A, Puzzolo D, Nowińska A, Wylegala E, Teper S (2014). Macular corneal dystrophy: In vivo confocal and structural data. Ophthalmology.

[20] Nowinska AK, Wylegala E, Teper S, Wróblewska-Czajka E, Aragona P, Roszkowska AM (2014). Phenotype and genotype analysis in patients with macular corneal dystrophy. Br J Ophthalmol.

[21] Siebelmann S, Scholz P, Sonnenschein S, Bachmann B, Matthaei M, Cursiefen C (2018). Anterior segment optical coherence tomography for the diagnosis of corneal dystrophies according to the IC3D classification. Surv Ophthalmol.

[22] Elhardt C, Priglinger SG, Karakolova Y, Mayer WJ, Wertheimer C (2019). Corneal dystrophies in optical coherence tomography. Ophthalmologe.

[23] Subaşı S, Yüksel N, Toprak M, Tuğan BY (2018). In vivo confocal microscopy analysis of the corneal layers in adenoviral epidemic keratoconjunctivitis. Turkish J Ophthalmol.

[24] Hou W, Sun X, Feng J, Zhang Y, Wang Z (2019). A 8-year retrospective clinical analysis of 272 patients of epidemic Keratoconjunctivitis in Beijing, China. BMC Ophthalmol.

[25] Kawamoto K, Chikama TI, Takahashi N, Nishida T (2009). In vivo observation of Langerhans cells by laser confocal microscopy in Thygeson’s superficial punctate keratitis. Mol Vis.

[26] Kobayashi A, Yokogawa H, Sugiyama K (2011). In vivo laser confocal microscopy findings of thygeson superficial punctate keratitis. Cornea.

[27] Donnenfeld ED, Cohen EJ, Ingraham HJ, Poleski SA, Goldsmith E, Laibson PR (1986). Corneal thinning in macular corneal dystrophy. Am J Ophthalmol.

[28] Dudakova L, Palos M, Svobodova M, Bydzovsky J, Huna L, Jirsova K (2014). Macular corneal dystrophy and associated corneal thinning. Eye.

[29] Feizi S, Karjou Z, Abbasi H, Javadi MA, Azari AA (2020). Characterization of In Vivo Biomechanical Properties in Macular Corneal Dystrophy. Am J Ophthalmol.

[30] Rubinstein Y, Weiner C, Einan-Lifshitz A, Chetrit N, Shoshany N, Zadok D (2016). Macular corneal dystrophy and posterior corneal abnormalities. Cornea.

[31] Akhtar S, Bron AJ, Hayes AJ, Meek KM, Caterson B (2011). Role of keratan sulphate (sulphated poly -N-acetyllactosamine repeats) in keratoconic cornea, histochemical, and ultrastructural analysis. Graefe’s Arch Clin Exp Ophthalmol.

[32] Yagi-Yaguchi Y, Yamaguchi T, Okuyama Y, Satake Y, Tsubota K, Shimazaki J (2016). Corneal higher order aberrations in granular, lattice and macular corneal dystrophies. PLoS One.

[33] Kamiya K, Kobashi H, Igarashi A, Shoji N, Shimizu K (2016). Effect of Light Scattering and Higher-order Aberrations on Visual Performance in Eyes with Granular Corneal Dystrophy. Sci Rep.

[34] Oshika T, Okamoto C, Samejima T, Tokunaga T, Miyata K (2006). Contrast Sensitivity Function and Ocular Higher-Order Wavefront Aberrations in Normal Human Eyes. Ophthalmology.

[35] Chuang J, Shih KC, Chan TC, Wan KH, Jhanji V, Tong L (2017). Preoperative optimization of ocular surface disease before cataract surgery. J Cataract Refract Surg.

[36] Epitropoulos AT, Matossian C, Berdy GJ, Malhotra RP, Potvin R (2015). Effect of tear osmolarity on repeatability of keratometry for cataract surgery planning. J Cataract Refract Surg.

[37] Javadi MA, Rafee’I AB, Kamalian N, Karimian F, Ja’farinasab MR, Yazdani S (2004). Concomitant keratoconus and macular corneal dystrophy. Cornea.

[38] Mohammad-Rabei H, Shojaei A, Aslani M (2012). Concurrent macular corneal dystrophy and keratoconus. Middle East Afr J Ophthalmol.

[39] Verma IC, Paliwal P, Singh K (2018). Genetic Testing in Pediatric Ophthalmology. Indian J Pediatr.

[40] Stone EM, Aldave AJ, Drack AV, MacCumber MW, Sheffield VC, Traboulsi E (2012). Recommendations for genetic testing of inherited eye diseases: Report of the American academy of ophthalmology task force on genetic testing. Ophthalmology.

[41] Liskova P, Veraitch B, Jirsova K, Filipec M, Neuwirth A, Ebenezer ND (2008). Sequencing of the CHST6 gene in Czech macular corneal dystrophy patients supports the evidence of a founder mutation. Br J Ophthalmol.

[42] Tuncay FY, Kurekci GK, Ergun SG, Pasaoglu OT, Akata RF, Dincer PR (2016). Genetic analysis of CHST6 and TGFBI in Turkish patients with corneal dystrophies: Five novel variations in CHST6. Mol Vis.

[43] Gruenauer-Kloevekorn C, Braeutigam S, Heinritz W, Froster UG, Duncker GIW (2008). Macular corneal dystrophy: Mutational spectrum in German patients, novel mutations and therapeutic options. Graefe’s Arch Clin Exp Ophthalmol.

[44] Moore CBT, Christie KA, Marshall J, Nesbit MA (2018). Personalised genome editing – The future for corneal dystrophies. Progress in Retinal Eye Research.

[45] Courtney DG, Moore JE, Atkinson SD, Maurizi E, Allen EHA, Pedrioli DML (2016). CRISPR/Cas9 DNA cleavage at SNP-derived PAM enables both in vitro and in vivo KRT12 mutation-specific targeting. Gene Ther.

[46] Christie KA, Courtney DG, Dedionisio LA, Shern CC, De Majumdar S, Mairs LC (2017). Towards personalised allele-specific CRISPR gene editing to treat autosomal dominant disorders. Sci Rep.

